# Intraoral Condition as a Risk Factor for Dysphagia in Non‐Hospitalized Older People: An Updated Systematic Review

**DOI:** 10.1111/scd.70253

**Published:** 2026-09-14

**Authors:** Ariyanti Rezeki, Lindawati S. Kusdhany, Muslita Indrasari, Czeresna Heriawan Soejono

**Affiliations:** ^1^ Department of Prosthodontics Faculty of Dentistry Universitas Indonesia Central Jakarta Indonesia; ^2^ Departement of Internal Medicine Faculty of Medicine Universitas Indonesia Central Jakarta Indonesia

**Keywords:** older people, oral health, oro motor function, oropharyngeal dysphagia, saliva

## Abstract

**Aim:**

To investigate the association between intraoral conditions and OD in community‐dwelling elderly populations to identify key screening indicators.

**Methods:**

A systematic search was conducted across PubMed, Scopus, and Cochrane databases for original research published between 2015 and 2025, following PRISMA guidelines. Inclusion criteria focused on studies involving subjects aged 65 years or older that examined the relationship between oral health status and OD. Methodological quality was assessed using the JBI critical appraisal checklist. The analysis methods and inclusion criteria were documented in a protocol published in the Prospective International Register of Systematic Reviews (PROSPERO) under the registration number CRD420261324418.

**Results:**

Fifteen articles met the inclusion criteria. All included studies demonstrated at least one positive correlation between an intraoral component and dysphagia. Key findings include; a reduced number of remaining teeth, lack of occlusal support, and poor functional tooth units (FTUs) were consistently associated with increased OD risk. Both subjective xerostomia and objective hyposalivation were linked to increased swallowing effort and impaired bolus transport. Reduced tongue strength, lip force, and buccinator muscle strength were identified as significant risk factors for sarcopenic dysphagia.

**Conclusion:**

Intraoral conditions play a critical role in the oral preparatory phase of swallowing. Establishing a standardized list of intraoral indicators—such as tooth count, salivary flow, and muscle strength is essential for health professionals to effectively screen for dysphagia risk during routine oral examinations.

## 1 Introduction

Dysphagia is defined as any alteration occurring in the process of swallowing, indicated by signs and symptoms affecting the mouth, pharynx, larynx, and/or stomach. It may involve a lack of coordination between respiratory and feeding systems, leading to aspiration pneumonia, dehydration, malnutrition, and increased mortality risk. Dysphagia may arise from various aetiologies, including neurological disorders such as dementia, Parkinson's disease, multiple sclerosis, stroke, head and neck cancer, cervical spine surgery, traumatic brain injury, and chronic obstructive pulmonary disease. Moreover, the ageing process is correlated with the onset of dysphagia. There are a few studies in the literature that have examined older individuals who are not hospitalized and do not have any severe diseases, yet show symptoms of swallowing difficulties. While numerous studies have investigated the relationship between intraoral conditions and swallowing disorders in elderly individuals with underlying comorbidities, research examining how these conditions affect dysphagia during healthy ageing remains a significant gap in the literature.

A compromised intraoral conditions will affect bolus formation in the swallowing phase, which can cause OD. Oral health refers to the condition of the teeth, gums, and the overall oral‐facial system that enables chewing, speaking, and smiling. Oral health is an important aspect of eating. Adequate masticatory function requires a sufficient number of healthy teeth or functioning prostheses for adequate occlusion, if it is not achieved then it will be difficult to comminute and digest the food, which results in impaired swallowing.

Mastication process that consists of grinding food with teeth, facilitated by the exertion of lingual, facial, and masticatory muscles, together with the presence of saliva. The food bolus must be adequately prepared to facilitate easy and safe swallowing. It must possess slippery, cohesive, and has plasticity properties to ensure the formation of a bolus of appropriate size and consistency that the tongue can transport efficiently to the oropharyngeal isthmus. Three key factors significantly impact masticatory function in elderly individuals: (i) dysfunction of the motor apparatus, (ii) the quantity and/or quality of saliva, and (iii) the number of natural antagonist teeth.

The impact of poor intraoral condition on masticatory function and eventually lead to malnutrition has been proven in the elderly. However, the scientific evidence assessing the influence of intraoral condition on OD without other underlyingsystemic factors in the elderly is limited. Identifying those parameters of poor oral health that increase the risk of OD is important for community‐dwelling elderly. Oropharyngeal dysphagiahas a major impact on the quality of life for the elderly, with physiological and social consequences. The main health complications for individuals are malnutrition, dehydration, and aspiration pneumonia, which lead to poor public health outcomes, such as increased length of hospital stay with readmissions, and increased morbidity and mortality. A wide range of oral health criteria are detailed in the literature. However, there is a need to define the relevant oral health criteria that could be used to signal those at risk of OD.

The objective of this systematic review was (i) to investigate the association between oropharyngeal dysphagia and intraoral conditions in non‐hospitalized older people, and (ii) to create a list of oral health indicators to enable dental and health professionals (physicians, speech therapists, nurses, caregivers) to screen older people for OD risk during oral examinations.

## 2 Materials and Methods

### 2.1 Protocol

This systematic review was carried out using the Preferred Reporting Items for Systematic Reviews and Meta‐Analysis Protocol (PRISMA‐P 2015) guidelines. The analysis methods and inclusion criteria were documented in a protocol published in the Prospective International Register of Systematic Reviews (PROSPERO) under the registration number CRD420261324418.

### 2.2 Study Eligibility Criteria

All articles including subjects aged 65 years or older, related to good or poor oral health status, which dealt with oropharyngeal dysphagia risk as the clinical outcome of interest were included. The study population was defined as non‐hospitalized older adults, encompassing both independent community‐dwellers and residents of stable long‐term care or nursing home facilities. To ensure the review reflected recent evidence, only original research published between 2015 and 2025 was selected for qualitative synthesis. References published prior to 2015 were utilized solely for theoretical and contextual purposes and were not included in the formal data extraction. During screening, studies whose titles and/or abstracts were related to subjects under the age of 65, and/or those dealing with the treatment of dysphagia or with methods of assessing dysphagia were excluded. Finally, patients in acute hospital settings with severe underlying diseases (e.g, recent stroke or head and neck surgery) were excluded to isolate the impact of intraoral conditions on healthy ageing.

### 2.3 Search Strategy

A comprehensive and systematic search strategy is employed to identify all relevant studies that meet the inclusion criteria. This strategy involves identifying appropriate databases, formulating precise search terms, and utilizing specific search techniques to maximize the retrieval of relevant literature. The following electronic databases are systematically searched: PubMed, Scopus and Cochrane. The main keywords used were “Dysphagia, Oral health, Elderly”. A combination of keywords and controlled vocabulary (MeSH terms in PubMed) related to both the exposure (intraoral conditions) and the outcome (dysphagia) is used. The search strategy was developed using combinations of keywords and Boolean operators as follows: (“Dysphagia” OR “oropharyngeal dysphagia”) AND (“oral health” OR “intraoral conditions” OR “saliva” OR “oromotor”) AND (“elderly” OR “older people”).

Filters were applied to restrict the search to English‐language publications and studies involving participants aged 65 years and older. Equivalent search strategies were adapted for each database according to their specific indexing systems and search functionalities. MeSH terms were used in PubMed, while free‐text keywords and title or abstract searches were applied in Scopus and Cochrane Library. Records were retrieved using the defined search strategy, and duplicate entries were removed. Studies were included if they involved individuals aged 65 years or above, addressed good or poor oral health status, and examined oropharyngeal dysphagia as the primary clinical outcome. During the screening process, studies were excluded if their titles and/or abstracts focused on populations younger than 65 years, or if they primarily addressed dysphagia treatment or methods for assessing dysphagia.

### 2.4 Data Items

A Microsoft Excel file was created, and a pre‐pilot form was tested by two investigators. All quantitative and qualitative variables were considered as items and were used as column headings while the articles themselves were listed in the rows. The articles did not necessarily contain all the items but were listed in those columns that were relevant. Finally, three groups of items were listed for data extraction: Tooth, saliva, and oromotor.

### 2.5 Methodological Quality Assessment (Risk of Bias)

Two investigators and one supervisor independently assessed the methodological quality of included studies using the JBI Critical Appraisal Checklist for Cohort Studies, an 11‐item tool evaluating: (1) similarity and recruitment of comparison groups, (2) comparability of exposure measurement between groups, (3) validity/reliability of exposure measurement, (4) identification of confounding factors, (5) strategies to address confounding, (6) freedom from the outcome at baseline, (7) validity/reliability of outcome measurement, (8) adequacy of follow‐up time, (9) completeness of follow‐up and reporting of loss to follow‐up, (10) strategies to address incomplete follow‐up, and (11) appropriateness of statistical analysis. Each item was rated “Yes,” “No,” “Unclear,” or “Not Applicable.” Disagreements between investigators were resolved through discussion until consensus was reached.

The percentage score for each study was calculated as the number of “Yes” responses divided by the total number of *applicable* items (i.e., excluding items rated “Not Applicable” from the denominator), multiplied by 100. Based on this percentage, studies were classified as high risk of bias (≤49% “Yes”), moderate risk (50%–69% “Yes”), or low risk (≥70% “Yes”). Consequently, these scores led us to interpret the findings as correlations rather than definitive causal relationships, underscoring the urgent need for standardized metrics in future research to strengthen the evidence‐based link between intraoral conditions and oropharyngeal dysphagia. These thresholds followed conventions commonly applied in prior systematic reviews using the JBI approach.

This appraisal directly informed how findings were interpreted. Because the majority of included studies were rated as moderate risk—driven primarily by inadequate identification or control of confounding factors (Item 4) and incomplete reporting of follow‐up procedures (Items 8–9)—associations reported in this review were interpreted as correlational rather than causal. Studies rated as low risk of bias (Lee, D.‐S. et al., Lu et al., and Wakabayashi H. et al.) additionally accounted for confounders, and their findings were given relatively greater weight in the narrative synthesis.

### 2.6 Data Synthesis

After analysis, the results were synthesized into a short narrative review, targeting the relationship between oral health and OD in an elderly population. The criteria that were used for this analysis are presented in tables.

## 3 Results

### 3.1 Study Selection

**FIGURE 1:**
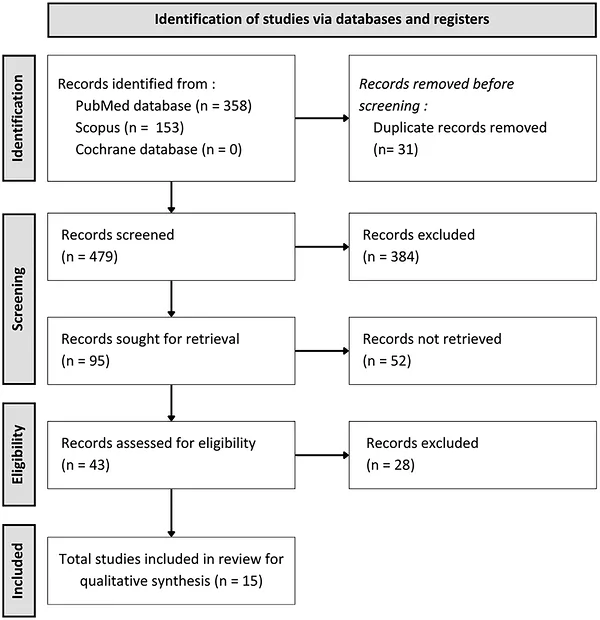
The flowchart for selecting studies.

A flow chart, presenting the selection process of included records, is displayed in Figure 1, following the PRISMA 2020 flow diagram guidelines. An advanced search was carried out on September 2025 using these terms for all available fields. Filters were applied to select English language texts and subjects aged 65 years or over. From PubMed database we collect 358 records, 152 from Scopus and no articles from the Cochrane Library. Records were collected using this search strategy, and 31 duplicates were excluded. Total of 479 articles were evaluated by title and 384 were excluded with a total of 95 records considered eligible for reading of the abstract. After analysis of the abstracts, a further 52 records were excluded based on the exclusion criteria. Of the 43 records that were eligible for full‐text reading, 28 were excluded at this stage. The final search for the qualitative synthesis included 15 articles, published between 2015 and 2025.

Risk of bias was assessed for all 15 included studies using the JBI Critical Appraisal Checklist for Cohort Studies (Table 1). Scores ranged from 54% to 72%. Most studies (*n* = 11) scored 64%, corresponding to moderate risk of bias, primarily reflecting insufficient reporting on confounding‐factor control and follow‐up completeness. Three studies—Lee, D.‐S. et al., Lu et al., and Wakabayashi H. et al.—achieved 72%, the highest score, placing them in the low‐risk category due to more complete confounder identification. Hägglund et al. [1] received the lowest score (54%), remaining within the moderate‐risk range but reflecting weaker reporting on statistical adjustment and follow‐up. No study met criteria for high risk of bias.

**TABLE 1:** The bias assessment using JBI critical appraisal checklist.

Author	Joanna Brigs Institute Items											Score (%)	Risk
	Q1	Q2	Q3	Q4	Q5	Q6	Q7	Q8	Q9	Q10	Q11		
Sakai K et al. 2019 [2]	Y	Y	N/A	Y	N	Y	Y	N/A	N	Y	Y	64%	Moderate
Indrasari M et al. 2024 [3]	Y	Y	N/A	Y	N	Y	Y	N/A	N	Y	Y	64%	Moderate
Dias R et al. 2024 [4]	Y	Y	N/A	Y	N	Y	Y	N/A	N	Y	Y	64%	Moderate
Lee, D.‐S et al. 2024 [5]	Y	Y	N/A	Y	Y	Y	Y	N/A	N	Y	Y	72%	Low
Lu et al. 2020 [6]	Y	Y	N/A	Y	Y	Y	Y	N/A	N	Y	Y	72%	Low
Shimazaki et al. 2020 [7]	Y	Y	N/A	Y	N	Y	Y	N/A	N	Y	Y	64%	Moderate
Brochier et al. 2018 [8]	Y	Y	N/A	Y	N	Y	Y	N/A	N	Y	Y	64%	Moderate
M Furuta et al. 2018 [9]	Y	Y	N/A	Y	N	Y	Y	N/A	N	Y	Y	64%	Moderate
M Takahashi et al. 2018 [10]	Y	Y	N/A	Y	N	Y	Y	N/A	N	Y	Y	64%	Moderate
Rech et al. 2017 [11]	Y	Y	N/A	Y	N	Y	Y	N/A	N	Y	Y	64%	Moderate
Wakabayashi H et al. 2017 [12]	Y	Y	N/A	Y	Y	Y	Y	N/A	N	Y	Y	72%	Low
Hägglund et al. 2017 [1]	Y	Y	N/A	Y	N	Y	Y	N/A	N	N	Y	54%	Moderate
Inui A et al. 2017 [13]	Y	Y	N/A	Y	N	Y	Y	N/A	N	Y	Y	64%	Moderate
Okabe Y et al. 2017 [14]	Y	Y	N/A	Y	N	Y	Y	N/A	N	Y	Y	64%	Moderate
Sakayori T et al. 2016 [15]	Y	Y	N/A	Y	N	Y	Y	N/A	N	Y	Y	64%	Moderate

*Note*: ^*^Y: Yes; N: No; N/A: Not Applicable.

### 3.2 Study Characteristics

The criteria used in the studies to establish relationships between dental status, saliva and oral motor skills and oral dysphagia are given in Table 2 respectively.

**TABLE 2:** The analysis of relationships between dental status, saliva and oral motor skills and oral dysphagia in studies included.

Article	Assessment of dysphagia	Assessment of Dental/Dryness/Oromotor	Relationship between dysphagia and Dental/Dryness/Oromotor
Sakai K et al. 2019 [2]	**Subjective: ‐** **Objective**: 100‐mL water swallow test (WST) while seated upright	‐ **Lip Force** using Lip de Cum, measuring forces acting in the vertical direction **‐ Tongue Strength** using balloon‐type oral probe ended instrument (JMS, Hiroshima, Japan), measuring strength of the front part of a tongue	Increased lip force or tongue strength was associated with a decreased likelihood of sarcopenic dysphagia [2].
Indrasari M et al. 2024 [3]	**Subjective**: Eating Assessment Tool in Indonesian EAT‐10 (EAT‐10‐ID) Questionnaire consisting of 10 items to identify the risk of experiencing dysphagia **Objective: ‐**	‐ Oral health‐related quality of life (OHRQoL) using The General Oral Health Assessment Index (GOHAI) questionnaire ‐ Occlusal tooth contact using the Eichner index ‐ Number of remaining teeth	The number of remaining teeth and occlusal tooth contact (Eichner index) had a significant relationship with the risk of dysphagia. Dysphagia and occlusal tooth contact are the risk factors most affecting OHRQoL [3].
Dias R et al. 2024 [4]	**Subjective**: Eating Assessment Tool in Indonesian EAT‐10 (EAT‐10‐ID) Questionnaire **Objective**: 3 oz water swallowing test	Dental examinations were carried out by one researcher recording the number of remaining teeth. The remaining teeth were defined as healthy, caries, restoration (including crowns, inlays, bridge abutment teeth), removable dentures that replace lost teeth.	The number of remaining teeth has a relationship with and influences the occurrence of dysphagia with an odds ratio of 4.318 [4].
Lee, D.‐S et al. 2024 [5]	**Subjective**: Dysphagia Risk Assessment Scale (DRAS) questionnaire **Objective:—**	Subjective: Masticatory discomfort reporting “difficulty chewing due to problems in the mouth” and pronunciation discomfort Objective: Clinical examination of unstimulated salivary flow rate, number of remaining teeth (excluding the third molar and exposed tooth roots), number of functional tooth units, orofacial muscle strength using the Iowa Oral Performance Instrument (IOPI)	The risk level of dysphagia was higher in groups who reported masticatory discomfort, pronunciations discomfort, had hyposalivation, less than 10 remaining teeth, who had 0–3 functional tooth units, weak anterior tongue elevation strength and weak buccinator muscle strength. Masticatory discomfort, hyposalivation, and weak buccinator muscle strength were identified as factors that increased the risk of dysphagia. [5]
Lu et al. 2020 [6]	**Subjective**: Ohkuma questionnaire consisting of 15 questions related to symptoms of dysphagia	Subjective: Xerostomia Inventory (condensed version) consisting of 5 items, which are (a) “My mouth feels dry when I eat a meal,” (b) “My mouth feels dry,” (c) “I have difficulty eating dry foods,” (d) “I have difficulty swallowing certain foods,” and (e) “My lips feel dry.”	The xerostomia group displayed more difficulty swallowing than the xerostomia‐free group did, the prevalence of dysphagia was significantly greater in those with xerostomia [6]
Shimazaki et al. 2020 [7]	**Objective**: Repetitive Saliva Swallowing Test (RSST)	Number of remaining teeth, periodontal status using community periodontal index (CPI), oral hygiene status using plaque index, dry mouth using a classification scale that included the condition of the oral mucosa	The odds ratio (OR) for swallowing difficulty was 3.42 among those among those who had 10–19 teeth without dentures and those who had 0–9 teeth without dentures, respectively, compared to individuals with ≥ 20 teeth without dentures. Individuals with moderate or severe dry mouth had statistically significantly higher OR (8.41) for swallowing difficulty than those without. Among dentate participants, those with abundant dental plaque showed a significantly higher OR (2.58) for swallowing difficulty compared to those with no or slight dental plaque [7].
Brochier et al. 2018 [8]	**Objective**: Clinical assessment of deglutition with two stages indirect swallowing test (saliva swallowing, forced coughing, anatomical conditions) and direct swallowing test (liquid, semi solid, solid), clinical signs of aspiration and cervical auscultation	Clinical exam by one dentist: Number of teeth, number of occlusal pairs, presence of dental prothesis (type, retention, stability, capacity to injure tissues, and aesthetics), xerostomia using Xerostomia Inventory (XI) with 11‐item rating scale representing the severity of chronic Xerostomia	‐ Older persons with no occlusal pairs had the highest prevalence of oropharyngeal dysphagia, when compared to older persons with 8 to 14 mixed pairs. ‐ Number of prostheses, prosthesis adaptation, and number of teeth were not associated with OD ‐ Older persons, who presented a highest score in the Xerostomia analysis, presented a high prevalence of oropharyngeal dysphagia [8].
M Furuta et al. 2018 [9]	**Objective**: Cervical auscultation through listening to the sounds of swallowing 3 mL water during pharyngeal phase	Number of remaining teeth (> 10 or < 9 remaining teeth) and usage of denture	Having many teeth and wearing dentures promoted normal swallowing function. Chewing difficulties resulting from having fewer teeth and no dentures can lead to dysphagia. [9]
M Takahashi et al. 2018 [10]	**Objective**: Sarcopenia using the diagnostic flow of the Asian Working Group for Sarcopenia, evaluating skeletal muscle mass reduction though calf circumference (CC) measurement; grip strength using a Jamar‐type digital hand dynamometer **Subjective**: Risk of dysphagia using the 10‐item Eating Assessment Tool	Number of remaining teeth, functional tooth units (FTU), Oral Health Assessment Tool (OHAT)	Patients with sarcopenia had significantly lower number of teeth, oral health status, and higher risk of dysphagia. [10]
Rech et al. 2017 [11]	**Objective**: Clinical evaluation of indirect swallowing test and direct deglutition test assessing the three food consistencies (pasty, liquid, and solid), cervical auscultation, clinical signs and symptoms of aspiration and penetration, anatomy and physiology (masticatory efficiency, etc)	Oral sensorimotor alteration evaluated by clinical examination of the lips (sealing, protrusion, retraction, diadochokinesia, strength, sensitivity), tongue mobility, tongue strength, tongue sensitivity, jaw, and larynx.	Individuals who had four or more oral sensorimotor alterations presented a higher prevalence of dysphagia. [11]
Wakabayashi H et al. 2017 [12]	**Objective**: 7‐point ordinal Dysphagia Severity Scale (DSS)	Occlusal support using the modified Eichner Index	Occlusal support was associated directly with dysphagia. Occlusal support affects both mastication and swallowing functions, as chewing movements are necessary to eat solid food [12].
Hägglund et al. 2017 [1]	**Objective**: The Swallowing Capacity Test (SCT) **Subjective**: The Swallowing Quality of life Questionnaire (SWAL‐QOL)	**Objective**: Clinical assessment including estimation of oral hygiene; numeric registration of teeth; the presence of bridges, partial denture, full denture, implants; the number of occluding surfaces; and a record of the need for dental care. **Subjective**: Questions about self‐perceived oral health, whether there was an established dental contact, and the time since the most recent dental visit	Better understanding of oral health in the older population studied will highlight the unaddressed needs related to oral care and malnutrition [1].
Inui A et al. 2017 [13]	**Subjective**: “Do you sometimes choke on drinks/food such as tea and soup?” (Yes / No) **Objective**: Repetitive Saliva Swallowing Test (RSST)	**Subjective**: Oral dryness (yes or no) **Objective**: Dental examination regarding teeth present were defined as healthy, carious or treated.	Individuals with oral dryness at risk of OD compared to others. Oral dryness and the number of teeth were found to be significantly related to dysphagia [13].
Okabe Y et al. 2017 [14]	**Objective**: Modified water swallowing test (MMSWT)	Oral examination carried out by 1 trained dentist including the number of teeth present (edentulous group, 1– 9 teeth, > 10 teeth) and posterior teeth occlusion (total number of functional tooth units/FTU)	The odds ratio (OR) of dysphagia risk decreased in subjects with higher total‐FTUs (OR ‐ 0.92) [14]
Sakayori T et al. 2016 [15]	**Objective**: Repetitive Saliva Swallowing Test (RSST) between 3 time points (before, at immediately after, and at 1 year after completion of the program)	**Objective**: Oral diadochokinesia where participants were instructed to pronounce /pa/, /ta/, or /ka/ for 5 s. This was performed between 3 time points (before, at immediately after, and at 1 year after completion of the program)	The average RSST score showed a decrease at 1 year after intervention but not statistically significant. Oral diadochokinesia (/pa/ and /ta/) showed a significant increase for all syllables upon completion of the program compared with at the beginning [15].

### 3.3 Results of Studies

#### 3.3.1 Association Between Oral Health Status and OD

Dental caries might indirectly contribute to dysphagia by causing pain and discomfort during chewing, which can lead to altered food choices and potentially affect bolus formation [16, 17]. The presence of caries was found to be higher in elderly patients with oropharyngeal dysphagia in one study. Periodontal disease, including gingivitis and periodontitis, has also been linked to dysphagia. Older patients with oropharyngeal dysphagia often present with a high prevalence of periodontal diseases [18]. The inflammation and potential tooth loss associated with periodontal disease could impact oral motor function and the overall health of the oral environment, potentially contributing to swallowing difficulties [13].

#### 3.3.2 Impact of the Number of Functional Teeth, Occlusion, and Chewing Function on OD

The oral health indicator most frequently assessed was the impact of the residual number of functional teeth or occlusion on OD. A significant association between teeth and OD was found in several studies. Cross‐sectional studies were often applied, so a causal relationship could not be established. A reduced number of teeth and edentulism have been consistently associated with an increased risk or severity of dysphagia in the elderly [4, 19, 20].

The ability to chew food effectively is dependent on the number of remaining teeth [21, 22]. Tooth loss can lead to difficulties in chewing and swallowing, potentially disturbing the coordinated execution of pre‐swallow and swallowing behaviors, particularly during the oral preparatory stage [23]. Studies have shown that elderly individuals with fewer functional teeth often report difficulties in both chewing and swallowing. A study involving a large sample of independently living elderly individuals found a positive correlation between the number of remaining teeth and maximum bite force, also reporting that individuals with fewer teeth were more likely to experience swallowing problems [22]. Tooth loss decreases chewing ability and can cause difficulties in forming the food bolus. It has been observed that bolus size tends to increase with a greater number of missing teeth, and larger boluses can interfere with optimal swallowing [21].

The role of dentures in mitigating the risk of dysphagia associated with tooth loss is complex [24]. Wearing dentures to maintain appropriate mandible position and proper occlusion has been suggested as important for smooth swallowing in older adults. Research indicates that individuals with fewer functional teeth or those not using dentures are more likely to experience subjective swallowing problems. However, the effectiveness of dentures in preventing swallowing problems may depend on their fit and regular use [24].

#### 3.3.3 Impact of Oral Dryness on Oral Dysphagia

Saliva plays a crucial role in the oral phase of swallowing. It provides lubrication, which is necessary for the smooth passage of food through the oral cavity and pharynx [6, 8]. Saliva also aids in bolus formation by moistening food particles and helping them cohere into a manageable mass. Furthermore, saliva contains enzymes that begin the process of digestion in the mouth. Adequate salivary production is therefore essential for efficient and safe swallowing [25].

Xerostomia, the subjective sensation of dry mouth, has been associated with increased perceived swallowing effort and difficulty. Individuals with higher levels of perceived mouth dryness report more effort during swallowing. Hyposalivation, the objective reduction in salivary flow rate, has also been linked to dysphagia. Reduced salivary secretion can impair bolus formation and oropharyngeal bolus transport, potentially leading to increased oropharyngeal residue.

Several factors contribute to reduced salivary flow in the elderly. The aging process itself can lead to changes in salivary gland structure and function. Many medications commonly used by older adults can have xerostomia as a side effect, contributing to reduced salivary flow. Systemic diseases prevalent in the elderly, such as Sjögren's syndrome and diabetes, can also affect salivary gland function.

## 4 Discussion

In this study, of the 15 publications examined, 15 studies demonstrated at least one positive correlation between an oral health component (dental, salivary, and/or oromotor function) and dysphagia.

Only two observational studies assessed the correlation between oropharyngeal dysphagia and oral state utilizing the three indicators of dental health, xerostomia, and oromotor function [5, 13]. The variety of oral health markers chosen for the 15 studies may impact the validity of the findings. Methodological heterogeneity in dysphagia assessment may also affect comparability between studies. Moreover, among the studies examining the correlation between oropharyngeal dysphagia and oral health outcomes, only 12 were structured to evaluate the strength of this correlation while adjusting for other variables [2, 3, 4, 5, 6, 7, 10, 11, 12, 14, 15] Consequently, while this review indicates a correlation between oral health and oropharyngeal dysphagia, it cannot establish causation.

The absence of scientific evidence may be attributed to the observational methodology employed in most studies and the unstandardized selection of oral health indices. For example, to assessed the relationship between mastication and oropharyngeal dysphagia, Eichner index and functional tooth unit are the most commonly used indicators, but other studies still use other methods. A multitude of oral health indicators were employed, and furthermore, the methodologies utilized for data collection exhibit significant variability.

Objective criteria were documented in 12 dental evaluation studies and in four studies evaluating oral motor skills [2, 5, 11, 15] Two of the research investigating the correlation between oropharyngeal and hyposalivation but only one included objective criteria [5, 7]. Moreover, the methodology employed for assessing both objective and subjective criteria was inadequately detailed in several studies. Consequently, further studies aimed at evaluating pertinent oral health indicators specifically associated with swallowing must be conducted. Future studies employing standardized and verified metrics are essential to establish evidence‐based correlations between oropharyngeal dysphagia and oral health.

Poor dentition, including tooth loss and ill‐fitting dentures, can significantly impair oral motor function, affecting mastication, bolus manipulation, and oral transit time [3, 4, 7, 8, 9]. Inefficient chewing due to a reduced number of teeth or poorly functioning dentures can lead to inadequately prepared food boluses that are larger and harder to swallow safely. This can prolong the residence time of food in the mouth and affect food choices [3, 8, 9, 14, 20, 25, 26, 27, 28].

Reduced salivary flow also plays a critical role in the development of dysphagia by affecting bolus formation and transport. Insufficient saliva hinders the formation of a smooth, cohesive bolus, making it harder to manipulate and propel through the oral cavity and pharynx. This can lead to increased effort during swallowing and potentially leave residue in the mouth and throat [5, 9, 20, 27, 28, 29, 30, 31, 32, 33].

Oral muscle strength and coordination are essential for effective swallowing.1 Weakness or incoordination of oral muscles, such as the tongue and buccinator muscles, can significantly impair the oral phase of swallowing. Studies have shown that weak buccinator muscle strength is independently associated with dysphagia risk in older adults [11, 15].

Changes in oral health can also affect sensory perception during swallowing. Effective bolus formation requires not only adequate mechanical breakdown of food but also proper mixing with saliva to ensure a cohesive and swallowable bolus. Taste alterations due to dry mouth and reduced tactile sensation can affect sensory feedback, potentially delaying the triggering of the swallow reflex and increasing the risk of silent aspiration. Poor oral hygiene may alter the oral microbiome and increase mucosal inflammation, which could additionally affect oral comfort and swallowing efficiency.

The findings of this review support a practical screening list for oropharyngeal dysphagia risk that can be proposed for use during routine oral examinations. The factors include dental factors, such as number of remaining teeth, inadequate posterior occlusal support or functional tooth units (FTUs), and the non‐use of dentures; salivary factors, including subjective xerostomia and objective hyposalivation; and oromotor factors, such as reduced tongue strength, diminished lip force, decreased buccinator muscle strength, and impaired oral diadochokinesia.

While the physiological mechanism between intraoral conditions and oropharyngeal dysphagia are likely universal, the generalizability of the findings is indeed influenced by healthcare access. Country‐specific factors, including healthcare systems and accessibility to dental care, may influence both oral health status and dysphagia prevalence. Most included studies were conducted in Japan, which may limit generalizability due to differences in healthcare accessibility, oral health practices, dietary patterns, and aging care systems across countries.

## 5 Conclusion

Poor dentition and reduced salivary flow is linked to the ability to adequately prepare the food bolus for swallowing. Effective bolus preparation, involving mastication and the mixing of food with saliva, is a critical step for safe swallowing. Impairments in this stage due to intraoral conditions significantly increase the risk of dysphagia. In conclusion, intraoral conditions are important contributors to oropharyngeal dysphagia risk in older people. Indicators such as tooth count, salivary flow, and muscle strength may support dysphagia risk screening during routine oral examinations.

## Conflicts of Interest

The authors declare no conflicts of interest.
